# The impact of the Mediterranean diet on immune function in older adults

**DOI:** 10.1007/s40520-024-02753-3

**Published:** 2024-05-23

**Authors:** Fiona Ecarnot, Stefania Maggi

**Affiliations:** 1 Department of Cardiology, University Hospital Besançon, Boulevard Fleming, Besançon, 25000 France; 2 SINERGIES Research unit, University of Franche-Comté, Besançon, 25000 France; 3 National Research Council, Neuroscience Institute, Aging Branch, Padova, Italy

**Keywords:** Mediterranean diet, Immunity, Gut microbiota, Vitamin, Polyphenol

## Abstract

Diet is one of the lifestyle factors that is most amenable to intervention, and has a substantial effect on the potential for successful aging and mitigation of the risk of disease. Good nutrition is a pillar of healthy aging, and a large body of evidence attests to the benefits of the Mediterranean diet on the quality of the aging process. The Mediterranean diet comprises a wide range of nutrients which, both individually and collectively, exert positive effects on immunity, in large part mediated by the gut microbiota. In this article, we review the effect of the Mediterranean diet on immunity, and how its beneficial effects are mediated by the gut microbiota. We review the effects of certain key components of the Mediterranean dietary pattern, including vitamins, zinc, selenium, and polyphenols. Overall, the existing body of evidence convincingly demonstrates that the Mediterreanean diet affects immune health by maintaining a healthy body weight and reducing the risk of metabolic and cardiovascular diseases; by reducing inflammation and by promoting a healthy gut microbiota profile.

## Introduction

Epidemiologists have long debated the limitations of the dominant causal models of diseases, which emphasize a linear and sequential view of causality. There has been increasing focus in recent years on the investigation of proximate, individual-level risk factors, and how social, environmental and biological factors jointly influence health in a lifelong approach. Indeed, aging is not a random process, but represents the convergence of numerous factors. A small proportion of changes with aging can be explained by genetic inheritance, but most of the variability in aging will depend on the broader characteristics of individuals and the environments they inhabit. Personal characteristics include factors such as sex and ethnicity, as well as occupation, educational attainment, and wealth. These in turn will contribute to social position and the ability to access resources. Combined with lifestyle and interactions with the environment, the culmination of all these characteristics will be each individual’s aging process [1]. Therefore, an ecological approach to health issues, and ecological models of intervention, have become distinctive features of disease prevention and health promotion in public health. Importantly, the factors that influence aging start to interact with each other from childhood onwards, and a significant proportion of the diversity in capacity and circumstance that is observed in older age is likely underpinned by the cumulative impact of person-environment interactions across the life course. Negative influences such as a sedentary lifestyle, poor diet, smoking and stress, are likely to accelerate the aging process, whereas virtuous behaviours such as exercise, appropriate nutrition, sleep (quality and quantity) and avoidance of stress, can help to promote decelerated aging [2].

One of the areas that is most amenable to intervention, with a view to positively shaping the aging process, is diet. Malnutrition is highly prevalent among older people, with reports of almost one quarter being affected by malnutrition, and up to 46% at risk of malnutrition among community-dwelling and institutionalized older adults [3, 4]. Conversely, obesity has also been shown to have negative consequences on the aging process, by increasing the risk of chronic disease and reducing response to certain vaccines (e.g. hepatitis B, tetanus, influenza and Covid-19), via such mechanisms as obesity-associated chronic low-grade inflammation or micronutrient deficiency [5]. A clear understanding of the natural history and physiological trajectories of normal biological systems, along with biological and social pathways, is needed when applying the life course approach, as these models suggest pathways linking exposures across the life course with later life health, and include the temporal ordering of exposure variables, their inter-relationships (directly or through intermediary variables) and the outcome measures. Good nutrition is a pillar of healthy aging, and a key target for interventions aimed at decelerating the aging process.

Inflammation is the natural reflection of the human immune system at work. The onset of inflammation in normal conditions is beneficial to the body, by responding to transient injury to promote repair and recovery. However, inflammation becomes problematic and deleterious when it persists, at a low-grade, in a chronic manner, leading to long-term damage across multiple systems. This chronic, low-grade inflammation, termed inflammaging [6], maintains elevated levels of biomarkers of inflammation in the circulation, and can be promoted by poor diet. This chronic, low-grade inflammation can also markedly increase the risk of several diseases, including diabetes, and coronary heart disease [7]. Muscle loss, which is a hallmark of sarcopenia, may compound chronic inflammation. The Western style dietary pattern is known to be one of the most powerful triggers of inflammation. The Western-like diet is characterized by frequent consumption of foods that are high in fats, cholesterol, simple sugars, processed food, “junk food” or “fast food”, additives, sugary beverages, and salt. Over time, it can lead to epigenetic reprogramming of innate immune cells, or “trained immunity,” inducing chronically augmented immune response that could potentially contribute to inflammatory disease [8]. A systematic review that included 46 studies found that meat-based or “Western-like” diets were positively associated with biomarkers of inflammation, notably C-reactive protein (CRP), while dietary patterns rich in fruit, vegetables and whole grains have inverse associations with inflammatory markers [9].

If we consider the aging process as the lifelong adaptation of the body to external and internal stressors, then the Mediterranean diet can be conceptualized, within this paradigm, as a form of chronic hormetic stress, whereby it will help to preserve a favourable balance between pro- and anti-inflammatory parameters, delaying biological aging and reducing or preventing age-related diseases [10]. Indeed, nutrition has been touted as the best kept secret in medicine, because its role in human health has been largely under-appreciated in research and clinical settings, even though it can be considered one of the most powerful tools that patients and physicians alike can wield to achieve better health [11]. Specifically, greater adherence to the Mediterranean diet has been shown to be associated with a wide variety of outcomes, including slower cognitive aging, lower mortality, lower incident frailty, and improvements in pain, disability and depressive symptoms [12–15]. Furthermore, a recent systematic review reported that adherence to the Mediterranean diet was associated with a positive effect on muscle mass and function, both key components of sarcopenia [16]. So how does the Mediterranean diet in particular act on our bodies to improve immune function and reduce the risk of disease?

The human immune system is a highly complex defence system, comprised of physical and biochemical barriers, as well as dedicated immune cells that are specialized in identifying, destroying and remembering invading pathogens. The first layer of non-specific innate immunity is of rapid onset (minutes to hours), and includes the physical barriers (such as the skin, mucous membranes, the naso-pharynx and respiratory tract), biochemical reactions (gastric acid, sweat, saliva….) and inflammation (inflammatory cytokines) [17]. The adaptive immune system, largely mediated by leukocytes, especially B- and T-cells, is slower to mount its response, but the response is highly specific and in some cases, may lead to lifelong protection against the invading pathogen [17].

The immune system is constantly at work to protect our bodies from exposure to external and internal insults. External challenges encompass all the challenges to the immune system that are encountered in the environment, such as bacteria, viruses, fungi and disease-causing pathogens of all sorts, physical injury, exposure to the sun or pollution, and – our particular focus here – food. As humans age, there is a gradual decline in immunity, with the result that older people are unable to mount as strong an immune response as their younger counterparts, rendering them more susceptible to infectious diseases (such as influenza or pneumonia) and to reactivation of latent infections (such as herpes zoster) [18].

The food we eat constitutes a substantial antigen load that must be dealt with by the human gut. The human intestine therefore plays a key role in human immunity, and the functioning of the gut and the immune system are intricately linked. There are hundreds of species co-existing in the gut microbiota, and a delicate balance must be maintained between commensal species that regulate gut homeostasis, and invading pathogens likely to cause disease or damage. Human immunity is largely modulated by the gut microbiota, which themselves are modulated by diet. Certain food groups, additives or ultraprocessed food products may disturb the equilibrium of the gut microbiota, leading to gut dysbiosis, which in turn may result in alterations to the gut barrier, activation of the immune system, or damage, such as breakdown, of the intestinal barrier, with leakage of toxins, bacteria or other substances into the circulation, with potential for damage to the brain, which is one of the pathways involved in the development of dementia [19]. Nutrition plays a key role in every stage of the immune response, and together with lifestyle factors, such as smoking, alcohol consumption, exercise, sleep and stress, determine the quality of immune function.

**Fig. 1:**
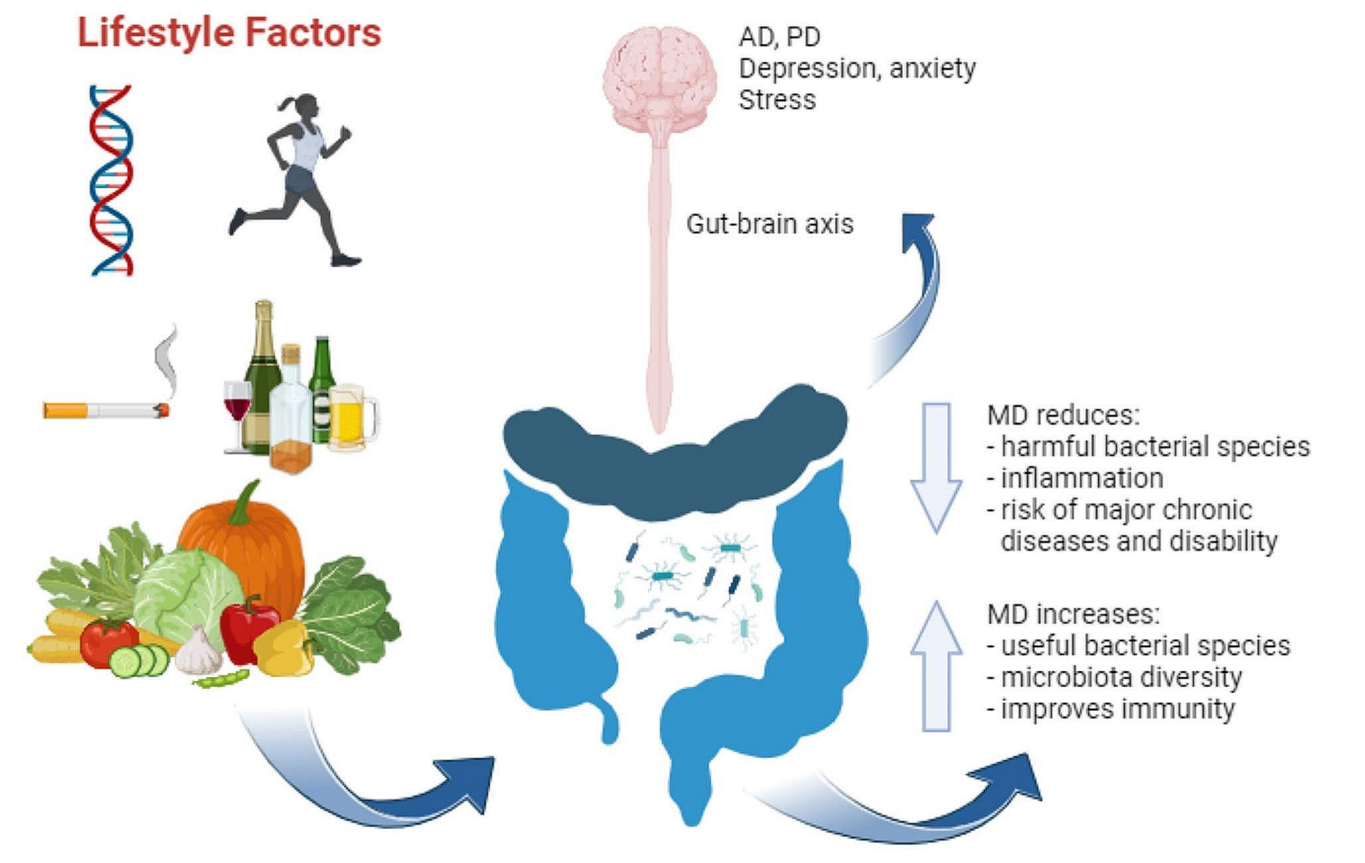
Mechanisms by which the Mediterranean diet can affect immune function. Genetics, lifestyle habits and diet combine to modulate the gut microbiota. The Mediterranean diet promotes the proliferation of beneficial bacterial species in the gut microbiota, thereby reducing inflammation and improving immune function. The effects of diet on the gut microbiota include modulation of intestinal epithelial permeability, which can allow toxic substances to travel via the gut-brain axis to the brain, where they may cause diseases such as Alzheimer or Parkinson’s disease, and affect depressive symptoms, anxiety and stress levels. Figure created with BioRender.com

Below, we review the Mediterranean dietary pattern, and how the various nutrients it provides can affect immune function and inflammatory status, especially in older adults (Fig. 1).

## Mediterranean diet

The Mediterranean Diet (MD) is actually better described as a dietary pattern, encompassing more than just food, but also values, such as the social aspect of dining in a convivial environment with family or friends, and an emphasis on sustainability by preferring seasonal and locally-produced foods. The MD is characterized by high consumption of fruit, vegetables, whole grains, legumes, nuts, and olive oil, with moderate consumption of fish and lean meat (poultry), and dairy produce, and only limited consumption of red meat, sweets and saturated fats. In 2010, the Mediterranean diet was added to the UNESCO Representative List of the Intangible Cultural Heritage of Humanity [20]. There is abundant evidence in the literature that adhering to the MD pattern is associated with a range of health benefits, including reduced risk of cardiovascular disease, cancer, diabetes and cognitive impairment [21, 22]. Moreover, the evidence stems from both Mediterranean and non-Mediterranean countries. While each individual component of the MD likely has beneficial effects in its own right, it is widely acknowledged that it is the combination of all the MD foods together in an overall dietary pattern that yields the positive health effects [22, 23]. By the same token, measuring or even distinguishing the impact of a single food on health outcomes or disease is almost impossible, because in a dietary pattern, there is exposure to a wide range of nutrients in varying quantities, with the attendant interactions and synergistic effects, which, together, provide benefits that go beyond the sum of the expected benefits from the individual components [24].

Below, we review how vitamins and polyunsaturated fatty acids, found in key components of the MD, such as fruits and vegetables, interact with the immune system, primarily via the gut and the microbiota.

## Vitamins

Vitamins play an essential role in both innate and adaptive immunity and across the whole life course.

**Vitamin E** is an umbrella term for a total of 8 tocopherols and tocotrienols present in food, with alpha tocopherol being the major isoform used by the human body. It is predominantly found in nuts and vegetable oils. Vitamin E is a potent and fat-soluble antioxidant that is present in cell membranes. It protects the polyunsaturated fatty acids (PUFAs) in the membrane against peroxidation, and thus, against the damage inflicted by free radicals and oxidative stress [25, 26]. It is one of the micronutrients with the most potent effect on immune function, and it has been shown in a prospective longitudinal cohort study of over 29,000 participants that higher levels of serum α-tocopherol at baseline were associated with lower risk of mortality [27].

**Vitamins A and D** contribute to maintaining the structural and functional integrity of mucosal cells, especially in the barriers (e.g. skin). They also promote normal functioning of immune cells such as natural killer (NK) cells, macrophages or neutrophils, while vitamin D promotes proliferation of immune cell subpopulations, and cytokines that fight against infection [17]. Vitamin D is also associated with an increase in expression of tight junction protein. Tight junctions between cells of the intestinal barrier maintain the structural integrity and prevent leakage, while breakdown of the tight junctions can lead to permeability of the intestinal barrier, allowing toxins, bacteria and other potentially harmful substances to enter into the circulation. Another important function of vitamin D is in calcium homeostasis, thereby contributing to bone health, and subjects with vitamin D deficiency have a higher risk of falls and fractures [28]. The precursors to vitamin A (carotenoids) are pigments found in fruit and vegetables, and have immunostimulant properties, activating cell signalling pathways. The MD is replete with fruit and vegetables that provide abundant carotenoids (e.g. leafy green vegetables, tomatoes, bell peppers, carrots, melons etc.). **Vitamin C** also promotes the integrity of epithelial barriers by enhancing the synthesis of collagen, and interacts with other antioxidant molecules, such as vitamin E, to restore them to their active state.

## Zinc

Zinc is a transition metal and an essential trace element required for essential physiological functions including growth, repair and metabolism. Zinc deficiency is common, especially in older individuals [29], and worldwide, it is a leading risk factor for bacterial pneumonia and diarrhoea [30]. Zinc plays a key role in the homeostasis of the immune system by promoting the development of Treg cells and suppressing proinflammatory lymphocyte differentiation [29]. Zinc also contributes to controlling oxidative stress and reducing inflammation through its effects on the function of several types of immune cells. Zinc has specific antiviral activity and has been shown to inhibit replication of coronaviruses, with increased susceptibility to COVID-19 reported among patients with low zinc levels [31]. The mechanisms underlying this effect may include its ACE 2 activity and zinc’s ability to affect tissue response to hypoxia through its participation in activating the transcription factor hypoxia-inducible factor 1α (HIF1α) [32, 33]. In a meta-analysis of 6 randomized trials totalling 2216 patients, zinc administered as an adjunct to the treatment of severe pneumonia was shown to be effective in reducing mortality [34]. In another systematic review and meta-analysis of 28 randomised controlled trials including a total of 5446 patients, Hunter et al. reported that zinc could prevent symptoms and shorten the duration of respiratory tract infection [35]. There is also some evidence to support that zinc levels are consistently significantly lower among patients with autoimmune diseases compared to controls, although the causal relationship between zinc and autoimmune disease, as well as the effect of zinc supplementation in reversing this trend remain to be confirmed [36].

## Selenium

Selenium is another essential trace element that also plays an important role in supporting the function of the immune system. Like zinc, it controls oxidative stress and inflammation. Selenium participates in a wide range of physiological processes, notably the regulation of antioxidant response to reactive oxygen species [37]. Both adaptive and innate (cell-mediated) immunity are affected by selenium, including such processes as inflammatory signalling capacity and the antipathogen activity of macrophages [38]. In a meta-analysis including 9 trials and totalling 220 selenium-treated individuals from North America and Europe, Filippini et al. found that overall, selenium supplementation did not substantially affect immunoglobulin or white blood cell concentrations, or cytokine levels [37]. The only notable result was an increase in natural killer cell lysis, but overall, the beneficial effects seems to be predominant only in subjects with low selenium levels. In line with this, selenium deficiency has been reported to be associated with increased susceptibility to viral infection due to the potent antioxidant effects of selenium [39, 40].

## Polyunsaturated fatty acids

Fats and oils are an essential part of the human diet. Dietary fatty acids include those that are loosely considered as “bad” fats (e.g. saturated fatty acids) and “good” fats (e.g. monounsaturated fatty acids and polyunsaturated fatty acids (PUFAs)). PUFAs can be subdivided into two major groups, namely omega-3 and omega-6 fatty acids, which are distinguished by the position of the double-bond in the carbon chain in their molecular structure [41]. The omega-3 PUFAs include notably alpha-linoleic acid, eicosapentaenoic acid, and docosahexaenoic acid, while the omega-6 PUFAs include linoleic acid and arachidonic acid [42]. PUFAs are found in vegetable oils (especially extra virgin olive oil), nuts, seeds and fish. Certain by-products resulting from the oxidation of PUFAs are known to be implicated in the pathogenesis of inflammation and diseases such as cancer, diabetes and atherosclerosis [43]. Conversely, the bioactive metabolites of omega-3 FAs, such as resolvins, protectins, and maresins, have anti-inflammatory properties [41, 42], pro-resolving lipid mediators which promote transition from a pro-inflammatory to an anti-inflammatory, pro-resolving state [44]. The anti-inflammatory effects of omega-3 PUFAs are exerted via several pathways including suppression of cytokine production, enhanced production of resolvins, or by affecting the properties of plasma membranes, which are rich in lipids. Furthermore, omega-3 PUFAs may promote efficient antigen presentation, thereby enabling faster immune resolution [41, 45]. They also prevent activation of the transcription factor Nf-kB, which plays a role in the onset of inflammatory reactions [10, 45]. Finally, omega-3 fatty acids are known to influence several cell populations involved in immunity (e.g. macrophages, monocytes, neutrophils and lymphocytes), augmenting phagocytosis and enhancing the removal of cell waste, and transitioning macrophages to an anti-inflammatory phenotype [45]. It has recently been proposed that the ratio of omega 6 to omega 3 fatty acids may be more important than the actual volume of either [46]. This is compounded by the Western-style diet, which has increased the omega-6/omega-3 ratio approximately 5-fold in the last few decades [47]. While the ideal ratio is around 4:1, a Western-style diet may increase this ratio to up to 20:1, thereby promoting inflammation [21]. This growing imbalance is largely due to increased intake of industrial, processed oils and lower consumption of fatty fish or shellfish [47]. This highlights the importance of the dietary pattern as a whole for health, rather than the individual contributions of specific nutrients.

## Gut microbiota

The diversity of microorganisms in the gut is essential to our overall health, by regulating inflammation and metabolism. Evidently, each person’s nutritional habits will influence the composition of their gut microbiome, which in turn modulates immune function as well as certain chronic diseases. The Mediterranean diet, with high consumption of fruit, vegetables and fiber, has been shown to be associated with increased microbial diversity [48]. Indeed, the gut microbiota in the average healthy adult comprises approximately 10 phyla in total, of which two are predominant (*Bacteroidetes* and *Firmicutes*). There are several highly represented taxa (e.g. *Bacteroides, Prevotella, Alistipes, Eubacterium*), and many others that are less represented, but which nonetheless play an important role in metabolism, such as taxa that can produce short-chain fatty acids (SCFAs) (e.g. *Faecalibacterium, Butyrivibrio, Succinivibrio, Ruminococcus*) [49, 50]. Depletion of SCFA-producing taxa is a hallmark of sarcopenic older adults [51]. There is a progressive change in the gut microbiota composition with increasing age, whereby biodiversity is reduced, and the balance between beneficial and deleterious species may be disturbed, rendering the individual less resilient to stressors [52]. This dysbiosis can lead to increased permeability of the intestinal barrier, allowing harmful substances such as lipopolysaccharides, toxins or cytokines, to cross into the circulation, promoting inflammation. These toxins may cross the blood-brain barrier and cause neural damage, in a key pathway leading to cognitive damage [19]. In this context, the influence of the diet on the gut microbiota is key. Fibers contain a wide range of polysaccharides that are not digestible by human enzymes, and represent a key substrate for depolymerization and fermentation of dietary fibers into SCFAs [53]. Conversely, a lack of dietary fiber can promote the proliferation of bacterial species that degrade the intestinal mucin layer, thus contributing to the breakdown of tight junctions between cells, and ultimately leading to intestinal permeability. Leaky gut is thought to be implicated in the initiation and progression of Alzheimer’s disease [54]. SCFAs produced by the microbiota promote the expansion and differentiation of regulatory T cells, which play a key role in maintaining immune homeostasis [55]. Overall, the available body of evidence supports the assertion that the Mediterranean diet promotes the proliferation of gut-healthy bacterial species, such as *Bifidobacterium*, *Lactobacillus*, *Faecalibacterium prausnitzii*, or *Akkermansia muciniphila*, which maintain the gut barrier function and regulate immune function. Conversely, the Mediterranean diet minimizes the proliferation of species such as *Ruminococcus* or *Bacteroides*, which may initiate and/or perpetuate inflammation. Therefore, greater adherence to the Mediterranean diet can improve obesity, inflammation and lipid profiles, and modulate the functioning of the host immune system.

## Polyphenols

Polyphenols are a family of water-soluble, plant-based molecules that are found abundantly in fruit, vegetables, herbs, spices, dark chocolate, tea and wine, for example. Polyphenols can be subdivided into categories including flavonoids, and non-flavonoids, which in turn can be subdivided into phenolic acids, polyphenolic amides, lignans and stilbenes. Growing epidemiological and research data concurs that a diet rich in polyphenols can provide protection against diseases as diverse as cancer, cardiovascular disease, diabetes, Parkinson’s disease, Alzheimer’s disease and asthma [56]. The beneficial activity of polyphenols is firstly dependent on their metabolism by the gut microbiota. Indeed, during digestion, polyphenols undergo a series of chemical enzymatic transformations that ultimately yield bioactive compounds that can then exert their beneficial effects [57]. Indeed, a small proportion of dietary intake of polyphenols is absorbed in the small intestine (estimated at < 10% [58]), while the remainder reaches the large intestine virtually intact and is metabolized there by the gut microbiota and rendered into bioavailable forms [59]. In the gut, polyphenols promote the proliferation of beneficial bacteria such as *Lactobacillus spp, Akkermansia spp. or Bifidobacterium spp*. by providing an energy substrate for their growth. Conversely, they can hamper the proliferation of species with harmful effects, e.g. *Clostridium spp* via bactericidal or bacteriostatic effect of phenolic compounds [59]. Accordingly, there is a bidirectional relationship between polyphenols and the gut microbiota, in that the microbiota transform the polyphenols into bioavailable compounds that can be distributed throughout the body, while the polyphenols in turn modulate the proliferation or suppression of specific species present in the gut [59]. Of note, inter-individual heterogeneity has been reported in the ability to metabolize polyphenols [60].

## Conclusion

In summary, it can be seen that the Mediterreanean diet affects immune health through a range of mechanisms. First, the Mediterranean dietary pattern can contribute to maintaining a healthy body weight, thereby avoiding obesity and its attendant harmful metabolic effects. Second, it can contribute to healthy aging by controlling risk factors for cardiovascular disease such as cholesterol and hypertension. Third, the individual components of the Mediterranean diet may directly control inflammation by direct actions on the various cell populations of the innate and adaptive immune system. Finally, the Mediterranean dietary pattern as a whole promotes a healthy gut microbiota profile, which in turn maintains immune homeostasis, minimizing the risk of leaky gut and passage of harmful toxins into the circulation, which may promote inflammation or chronic disease. In this context, if “food is medicine”, then surely the Mediterranean diet is the best medicine of all.

## Data Availability

No datasets were generated or analysed during the current study.
